# Cleavage Fracture of the Air Cooled Medium Carbon Microalloyed Forging Steels with Heterogeneous Microstructures

**DOI:** 10.3390/ma15051760

**Published:** 2022-02-25

**Authors:** Gvozden Jovanović, Dragomir Glišić, Stefan Dikić, Nenad Radović, Aleksandra Patarić

**Affiliations:** 1 Institute for Technology of Nuclear and Other Mineral Raw Materials, Metallurgical and Environmental Engineering, Bulevar Franše d’Eperea 86, 11000 Belgrade, Serbia; a.pataric@itnms.ac.rs; 2 Faculty of Technology and Metallurgy, University of Belgrade, Karnegijeva 4, 11120 Belgrade, Serbia; gile@tmf.bg.ac.rs (D.G.); nenrad@tmf.bg.ac.rs (N.R.)

**Keywords:** cleavage fracture stress, medium carbon forging steel, microalloyed steel, acicular ferrite, heterogeneous microstructure

## Abstract

Cleavage fracture of the V and Ti-V microalloyed forging steels was investigated by the four-point bending testing of the notched specimens of Griffith-Owen’s type at −196 °C, in conjunction with the finite element analysis and the fractographic examination by scanning electron microscopy. To assess the mixed microstructure consisting mostly of the acicular ferrite, alongside proeutectoid ferrite grains and pearlite, the samples were held at 1250 °C for 30 min and subsequently cooled instill air. Cleavage fracture was initiated in the matrix under the high plastic strains near the notch root of the four-point bending specimens without the participation of the second phase particles in the process. Estimated values of the effective surface energy for the V and the Ti-V microalloyed steel of 37 Jm^−2^ and 74 Jm^−2^, respectively, and the related increase of local critical fracture stress were attributed to the increased content of the acicular ferrite. It was concluded that the observed increase of the local stress for cleavage crack propagation through the matrix was due to the increased number of the high angle boundaries, but also that the acicular ferrite affects the cleavage fracture mechanism by its characteristic stress–strain response with relatively low yield strength and considerable ductility at −196 °C.

## 1. Introduction

**Figure 1:**
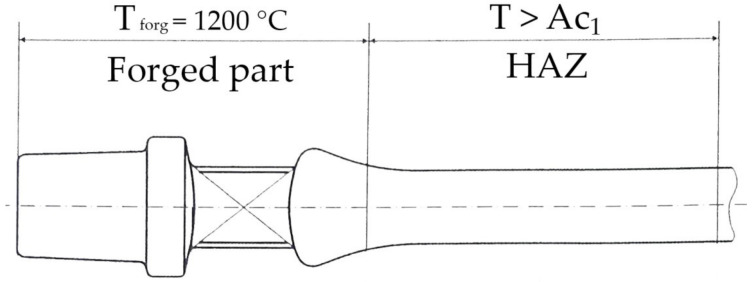
Schematic illustration of heat affected zone in the forged rod.

Medium carbon microalloyed forging steels have been introduced into forging practice with the intent to replace costly procedures of quenching and tempering. Despite the fact they are high in strength, in some cases forging steels lack the desired impact toughness up to 40%, as shown in [1]. Forged steel parts are usually produced by heating to a high temperature and forged to the desired shape. In the case of the drilling rods for the petrol industry, the rod’s end is heated with an induction heater to about 1200 °C and forged into a suitable shape for subsequent machine working, as shown in Figure 1. Consequently, the neighboring part of the rod is heated above Ac_1_ temperature by conduction, therefore forming a heat affected zone (HAZ), fully analogous to the HAZ in a welding process.

By continuous cooling from the forging temperatures, a range of heterogeneous microstructures can be formed. Depending on the cooling rates and the chemical composition, microstructure of the medium carbon microalloyed steel could consist of ferrite, perlite, bainite, acicular ferrite, martensite, and retained austenite [2,3]. The same heterogeneous structures can be formed in HAZ both in the case of welding and forging, except for the differences in the content of each microconstituent and the size proportions of the HAZ.

Previous research have noted the positive influence of acicular ferrite (AF) in the microstructure on the toughness of low carbon and welded pipeline steels [4,5,6,7,8,9,10]. AF is formed by bainitic reaction, but unlike bainite that grows as a sheaf of ferrite plates from the austenite grain boundary, AF nucleates intragranularly at inclusions, forming fine interlocking structure of laths and/or plates [11,12]. It is believed that AF increases toughness by forcing propagating crack to deflect at the high angle boundaries between the ferrite laths and plates [13]. Formation of AF is also observed in medium carbon microalloyed steels, coexisting with other microconstituents, primarily ferrite and pearlite, in continuously cooled samples from the temperature of austenitization or hot working [3,14]. Promising results in improving the toughness of the medium carbon microalloyed steels have been noted in those with a predominantly acicular ferrite structure [4,15,16,17]. For continuously cooled medium carbon microalloyed steels fracture studies were focused mainly on ferrite-pearlite and bainite structures [18,19], and it was found that in general, cleavage fracture was initiated or controlled by the broken coarse TiN particles in the zone of high stress in front of the notch in the four-point bending specimens [20,21,22]. Effective surface energy for those steels has been determined to be less than 50 Jm^−2^ at −196 °C [20,23]. However, little attention has been given to the cleavage fracture in continuously cooled medium carbon microalloyed steels with the predominantly acicular ferrite structure.

The aim of this work is to investigate the influence of a classical heterogeneous structure predominantly made up of acicular ferrite on the micromechanical mechanism of cleavage fracture for two different medium carbon microalloyed steels. An attempt is made to better understand how the control of a heterogeneous structure (i.e., acicular ferrite) influences fracture behavior, similar to approaches in the studies of other multiphase steels, such as TrIP (transformation induced plasticity), TwIP (twinning induced plasticity), or DP (dual phase) steels [24].

## 2. Materials and Methods

The chemical compositions of two commercial medium carbon microalloyed steels are given in Table 1. The Ti-V and V microalloyed steel were received as hot-rolled rods 22 mm and 19 mm in diameter, respectively. In order to eliminate rolling texture, the as-received rods were homogenized at 1250 °C for 4 h in an argon atmosphere, followed by quenching in oil at room temperature. In order to achieve predominantly acicular ferrite structure by continuous cooling, specimens were afterward reaustenitized at 1250 °C for 30 min in argon and then cooled at still air.

Metallographic specimens were cut from the rods in a transverse direction and prepared for light microscopy examination by grinding, polishing, and etching in 2% solution of nitric acid in ethanol. The microstructure of the steels was examined by light microscopy, and the micrographs were captured by using a digital camera. Quantitative analysis of the microstructures was performed using FIJI software [25]. A separate set of specimens was prepared for measurement of the previous austenite grain size (PAGS). In order to reveal austenite grain boundaries, specimens were reheated to 1250 °C for 15 min, water quenched, subsequently tempered at 450 °C for 24 h, cooled in still air to room temperature subsequently etched in a saturated solution of picric acid with 1 cm^3^ of HNO_3_. Average values of PAGS measured by the linear intercept of the grain boundary segregations network were 80 ± 10 µm for Ti-V steel and 100 ± 10 for V steel.

In order to investigate the cleavage fracture of the steels four-point bending (4PB) tests were carried out at the temperature of liquid nitrogen (−196 °C) with a constant crosshead speed of 0.1 mm min^−1^. Four-point bending notched specimens are the same type as those used by Griffiths-Owen [26].

The finite element modeling (FEM) was performed using SIMULIA Abaqus software in order to calculate stresses and strains in the four-point bending (4PB) specimens. One-quarter of the specimen was modeled in three dimensions. Hexagonal eight-node element type with reduced integration was used (C3D8R) with geometric nonlinearity taken into account. The mesh was generated so that the size of the elements is gradually refined toward the notch tip, from 0.7 to 0.07 mm. Displacement controlled loading was applied with the maximum displacement that corresponds to the fracture load reached in the experiment. Elastic-plastic response of the steels was modeled by the elastic modulus and Poisson’s ratio of 200 GPa and 0.28 [26], respectively, and by using the true stress–strain curves constructed by polynomial regression of the experimental data obtained by the uniaxial tension testing at −196 °C.

The stress–strain dependence was determined by uniaxial tensile testing in a liquid nitrogen bath at a constant crosshead speed of 0.1 mm min^−1^, which provides an initial strain rate of the same order of magnitude as in the four-point bending test (≈10^−5^ s^−1^). Uniaxial tensile testing was performed using proportional cylindrical specimens 6 mm in diameter and 30 mm in gauge length (EN ISO 6892-1).

Fracture analysis was performed using a scanning electron microscope (SEM) equipped with an energy dispersive X-ray spectrometer (EDS). Fracture origins were determined by tracking the markings on the cleavage facets. Distance of the fracture initiation site from the notch root, X_0_, and the size of the initial cleavage facet was measured at the SEM micrographs. The cleavage facets were approximated by an ellipse with major axes corresponding to the maximum and minimum ferret diameters of the facet (D_max_ and D_min_, respectively).

The local cleavage fracture stress, σ_F_^*^, was determined from the FEM calculated maximum principal stress distribution at the distance of the cleavage initiation site from the notch root, X_0_. Critical cleavage fracture stress, σ_F_^*^, and the first cleavage facet dimensions are related by Griffith’s equation [18,19]:

(1) $$\sigma_{F}^{*}=\sqrt{\frac{\pi \cdot E\cdot \gamma }{\left(1-\nu^{2}\right)\cdot D_{\mathrm{eff}}}}$$

where γ is the effective surface energy, D_eff_ is the effective diameter of the first cleavage facet, E is the modulus of elasticity, and ν is the Poisson’s coefficient. In this paper, effective diameter, D_eff_, was calculated by the following formulas [17,18]:

(2) $$D_{\mathrm{eff}}=\frac{D_{\min}}{\phi^{2}}\frac{\pi^{2}}{4}$$

(3) $$\phi =\frac{3\pi }{8}+\frac{\pi }{8}{\left(\frac{D_{\min}}{D_{\mathrm{eff}}}\right)}^{2}$$

Based on Equation (1), the effective surface energy, γ, was determined from the plot of the local critical cleavage fracture stress versus the reciprocal square root of the first cleavage facet effective diameter, *σ*_F_^*^-D_eff_^−1/2^

## 3. Results and Discussion

### 3.1. Microstructure

**Figure 2:**
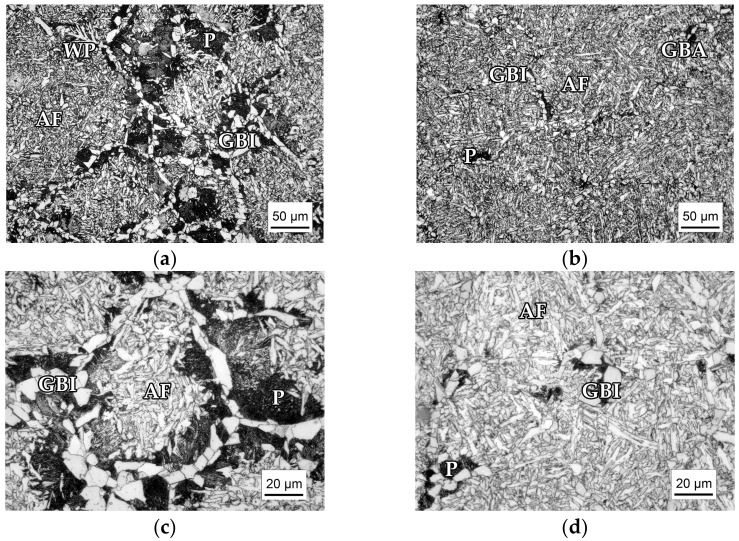
Optical micrographs of the medium carbon V and Ti-V microalloyed steels samples air-cooled from the austenitization temperature of 1250 °C: (**a**) Overall microstructure of the V microalloyed steel; (**b**) overall microstructure of the Ti-V microalloyed steel; (**c**) details of the V steel microstructure;(**d**) details of the Ti-V steel microstructure. P-pearlite, GBI-grain boundary idiomorphs, GBA-grain boundary allotriomorphs, AF-acicular ferrite, WP—Widmanstätten side plates.

Microstructures of the steels investigated, shown in Figure 2, consist of ferrite, pearlite, and acicular ferrite. The steel with V as the main microalloying addition (“V steel”) is characterized by the continuous network of proeutectoid grain boundary ferrite (GBF) along the previous austenite grain boundaries bordered by the pearlite nodules (P). Grain boundary ferrite consists of both elongated allotriomorphs (GBA) and polygonal idiomorphs (GBI). Most of the previous austenite grain interior is occupied by acicular ferrite (AF), mainly separated from the GBF by the perlite (P), as can be observed in Figure 2a,c. Unlike the V steel, in the structure of the steel microalloyed with Ti and V (“Ti-V steel”) proeutectoid ferrite grains and pearlite are sparse and discontinuous, while acicular ferrite occupies an almost complete volume of the previous austenite grain (Figure 2b,d). Estimated volume fractions for V steel are 70% of AF and 20% of P, while for steel, it is 96% AF and only 2% P, and the rest is GBF.

Small isolated islands of pearlite alongside a discontinuous network of GBF indicate markedly higher hardenability of the Ti-V steel. Hardenability in terms of retardation of diffusional transformation of austenite can be rationalized in terms of the chemical compositions of the steels. Besides higher carbon content, the higher hardenability of Ti-V steel is attributed to the higher content of substitutional alloying elements—Cr, Mo, and Ni. Manganese content, which is known to have a strong retarding effect on the diffusional decomposition of austenite [27], is essentially equal for both steels. However, a possible influence of free excess vanadium atoms in austenite solid solution that are not tied in V(C,N) particles should be taken into consideration. Vanadium atoms in solid solution increase hardenability by segregating to austenite grain boundaries, rendering them energetically less suitable for nucleation of proeutectoid ferrite [6,28]. The distribution of the microalloying elements together with the temperatures for the complete dissolution of VN and VC particles is given in Table 2. Considering temperatures for complete dissolution calculated using equations for solubility products [29]:

(4) $$log\left[V\right]\left[N\right]=-7840/T_{VN}+3.02$$

(5) $$log\left[V\right]\left[C\right]=-9500/T_{VC}+6.72$$

it follows that VN and VC carbides were completely dissolved at the austenitization temperature of 1250 °C. We can assume that the total amount of titanium is precipitated as TiN particles due to low solubility even at the austenitization temperature [19].

The distribution of the microalloying elements was calculated by taking the stoichiometric ratios in TiN and VN particles of Ti:N = 3.4, and V:N = 3.6, with the assumption that upon cooling from the austenitization temperature, precipitation of VN particles takes precedence over the precipitation of VC particles. From these considerations, it follows that higher concentrations of free vanadium atoms can be expected in Ti-V steel, and consequently, that it would impose a stronger suppressing effect on diffusional transformations of the austenite.

The influence of the prior austenite grain size (PAGS) on hardenability is related to the number density of potential grain boundary nucleation sites [30]. The addition of Ti in the Ti-V steel contributes to grain size control and austenite grain refinement [13,31]. Austenite grain size in both steels examined was relatively large, about 80 μm in Ti-V steel and 100 μm in V steel, which is favorable for the intragranular nucleation. The addition of Ti was in order to prevent grain growth of austenite at forging temperature, i.e., no influence on hardness was expected. The difference in PAGS did not impose a noticeable effect on the hardenability of the steels; thus, higher hardenability of the Ti-V steel implies the dominant influence of the alloying elements in solid solution, especially free excess V.

Transformation on continuous cooling from the austenitization temperature begins with proeutectoid ferrite formation at austenite grain boundaries, advances by the formation of the pearlite, and finishes by bainitic reaction at lower temperatures when diffusional transformations are no longer possible. Nucleation of the bainite at the austenite grain boundaries is precluded by the presence of grain boundary ferrite grains, mostly in V steel, or due to segregation of alloying elements, presumably free excess V in Ti-V steel, and therefore intragranular nucleation of acicular ferrite takes place. Intragranular nucleation is promoted by the presence of second phase particles suitable as ferrite nucleation sites. VN particles precipitated at MnS inclusions seem particularly effective as intragranular ferrite nucleation site [32,33]. Considering the chemical composition of the steels investigated, complex VN/MnS particles could be expected as the main acicular ferrite nucleation site, although other particles with MnS core, such as CuS and TiN should not be excluded [20,34,35,36,37].

AF is considered as an effective barrier for crack propagation due to the high density of high angle boundaries by forcing propagating cracks to change direction frequently, thus increasing the overall energy needed for fracture [6]. However, previous investigations established two distinct AF morphologies, generally defined in analogy to bainite, depending on the temperature of isothermal transformation, as upper and lower AF [38]. Lower AF formed at temperatures around 400 °C is depicted by the sheaves of nearly parallel plates or laths with similar crystallographic orientation, while upper AF formed at around 450 °C is characterized by the fine interlocking structure of ferrite plates/laths with high crystallographic misorientation [4,12,39]. Therefore, it seems rational to consider acicular ferrite obtained by continuous cooling as a mixture of both isothermal types, although it cannot be easily discerned at the light microscopy level.

### 3.2. Fractography

**Figure 3:**
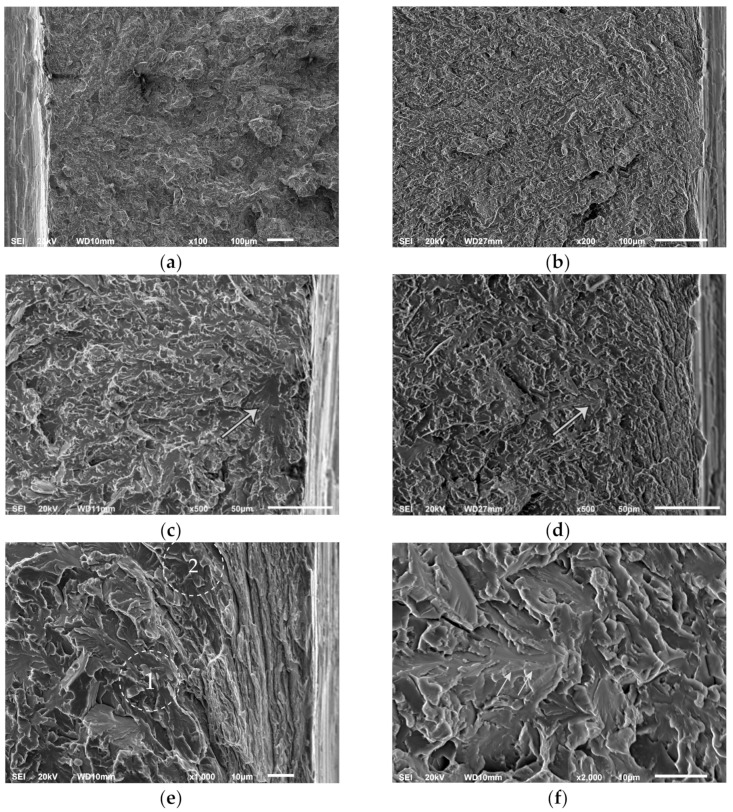
SEM micrographs of the fracture surfaces: (**a**) Macroscopic markings pointing to the fracture initiation site in V steel; (**b**) macroscopic markings pointing to the fracture initiation site in Ti-V steel; (**c**) coarse facets near the fracture origin in V steel sample; (**d**) fracture origin in Ti-V steel sample and an example of the tilted boundary between two coarse cleavage facets nearby; (**e**) typical cleavage initiation site in V steel, marked with number 1; (**f**) coarse facets separated by the boundary between the two grains with tilted crystallographic orientation near the fracture origin in Ti-V steel.

Macroscopic V-shaped markings at the fracture surface (“chevron markings”) points to the origin of the fracture located near the notch root (Figure 3a,b). The fracture surface is characterized by the fine irregular cleavage facets (Figure 3c,d) alongside with the islands of coarse facets. These isolated coarse facets are also irregularly shaped and more often present in the Vsteel than in the Ti-Vsteel (Figure 3c,d). Observed fracture surface features could be related to the underlying structure consisting of various ratios of grain boundary ferrite, pearlite, and acicular ferrite. In that manner, large facets may be correlated with the fractured grain boundary ferrite grains and/or coarse pearlite nodules, while small irregular facets correspond to the fractured AF plates.

The appearance of the fracture surface reflects the underlying microstructures of the two steels examined. Therefore, in the Ti-V steel with the predominantly AF structure and lower volume fraction of GBF and pearlite, there are fewer coarse cleavage facets, which are generally smaller in size than in the V steel specimens. An example of coarse facets or a group of facets is shown in Figure 3c,d for the V and Ti-V steel, respectively. At small facets, fracture marks are barely visible and could be considered as feather markings rather than river lines, which makes them difficult to trace back to the origin of the fracture. Pronounced tearing lines bordering fine facets seem to indicate an effect of the high-angle crystallographic misorientation between individual acicular ferrite plates, forcing propagating crack frequently to deflect. An example of the group of coarse facets separated by the ridge that represents the boundary between adjacent grains with tilted crystal orientations is shown in Figure 3f. Such coarse facets could be associated not only with individual GBF and pearlite but also with the aggregates of ferrite and pearlite with similar or the same crystallographic orientation [40,41]. However, cleavage fracture traces bypass the facets in this example, shown in Figure 3f, and therefore could not be the origin of the cleavage fracture. Additionally, having in mind that continuously cooled structures of the medium carbon microalloyed steels may contain both upper and lower acicular ferrite, it could be assumed that some of the coarser facets correspond to the sheaves of acicular ferrite plates with similar crystallographic orientation. In this respect, previous studies have indicated that microstructural units controlling cleavage fracture in an acicular ferrite structure could be a group of ferrite plates with crystallographic misorientation below 15° [13,42].

**Figure 4:**
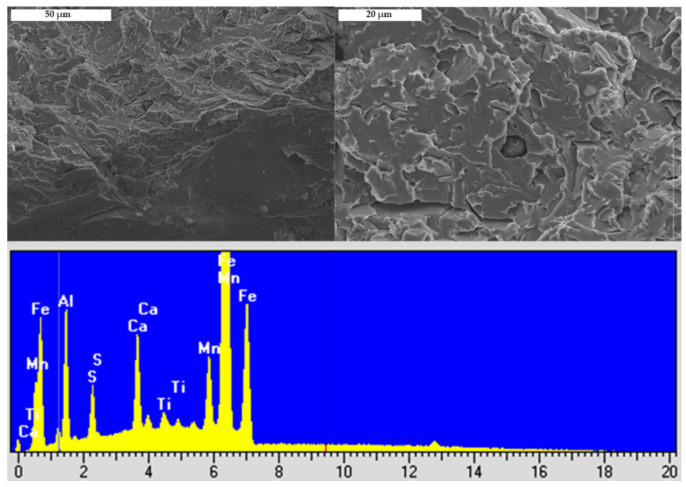
SEM micrographs and the EDS spectra of the particle found near the notch of the 4PB specimen.

**Figure 5:**
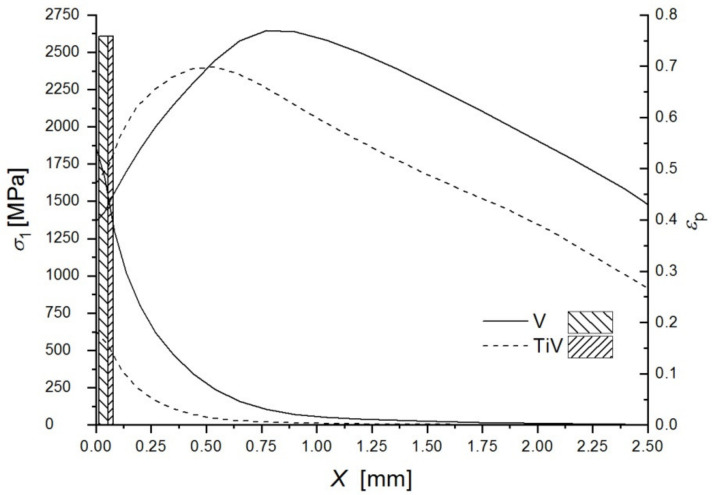
Maximum principal stress and the plastic strain distribution along the distance from the notch root *X*_o_ of the 4PB samples for the V and Ti-V microalloyed steel at the cleavage fracture initiation. Shaded boxes indicate the zone of the cleavage fracture initiation.

In both steels fracture originates from the area almost at the notch root. There are not any fractured second phase particles or inclusions at the cleavage fracture origins in any of the samples of both steels. SEM micrograph with EDS in Figure 4 shows spherical MnS-based complex particle found near the cleavage initiation site. This particle is not broken, fine cleavage markings on the surrounding facets bypass it, and therefore not related to the fracture initiation. By following river lines and feather markings, a facet or a group of facets at the initiation sites near the notch root were discovered in both steels. Distances measured from the notch root to the cleavage initiation sites are between 14 and 56 μm for V steel specimens and 56 to 131 μm for the Ti-V steel specimens (Table 3). According to the distribution of the maximum principal stress, σ_11_, calculated by the FEM, shown in Figure 5, peaks of the maximum principal stress reach approximately 2300 MPa and 2500 MPa for V steel and Ti-V steel, respectively. Cleavage fracture in microalloyed steels with ferrite-pearlite, bainite, or martensite structures is generally initiated by the fracture of a coarse second phase particle. In the vast majority of the cases, it was TiN particle, 26 μm in diameter, in the zone of the peak value of the maximum principal stress [18,20,23,43,44]. It could be concluded that in this case, with the predominantly AF structure, an alternative mechanism of cleavage initiation was active. A similar case of cleavage initiation was also found near the notch of the 4PB. Therefore, it could be concluded that the local stress did not attain the critical level for the fracture of coarse TiN particles. Furthermore, from the graphs shown in Figure 5, it is clear that all the fracture origins are located within the narrow zone of high plastic deformations.

Cleavage fracture initiation by the Smith’s mechanism, involving fracture of the cementite plate at the ferrite grain boundary [45,46] also does not seem probable in this case, at the stress levels present in the vicinity of the notch. Taking the value of effective surface energy for cementite of 9 Jm^−2^ [47] and by using Griffith’s equation for the thru-thickness crack:

(6) $$\sigma_{F}^{*}=\sqrt{\frac{4E\gamma }{\pi \left(1-\nu \right)C}}$$

it can be calculated that the highest stress values at the locations of the fracture initiation given in Table 3, were not sufficient for the propagation of the cracks nucleated at the cementite plates thinner than approximately 0.7 μm. This consideration leads to an assumption that initial microcracks were formed by the rupture of the grains under the high plastic deformations. In fractographs in Figure 3d,e, on the right side, many ruptures formed at the notch root are seen. Facet in Figure 3e marked as number 2 was formed almost at the notch root but had not been a part of the main propagating crack front. Bearing in mind the role of the high plastic strains, a mechanism of damage accumulation in pearlite by fracture of multiple cementite lamellas (Miller–Smith mechanism) could be considered as a plausible mechanism of the cleavage crack nucleation, in analogy to the cleavage fracture initiation in the pearlitic steels [48,49]. It could be assumed that microcracks formed at the pearlite nodules would easily propagate through the low angle boundary with neighboring proeutectoid ferrite. A relatively large microcrack formed by this mechanism could easily propagate at relatively low stresses near the notch tip.

**Figure 6:**
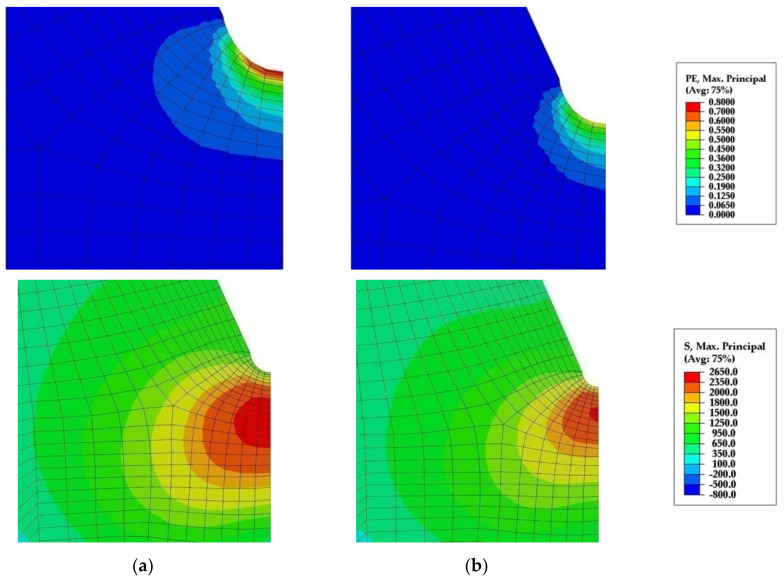
Contours of the plastic strain (above) and the maximum principal stress(below) distribution in front of the notch root of the 4PB specimen at the cleavage fracture initiation from the same viewpoint in FEMfor the (**a**) V steel; (**b**) Ti-V steel.

**Figure 7:**
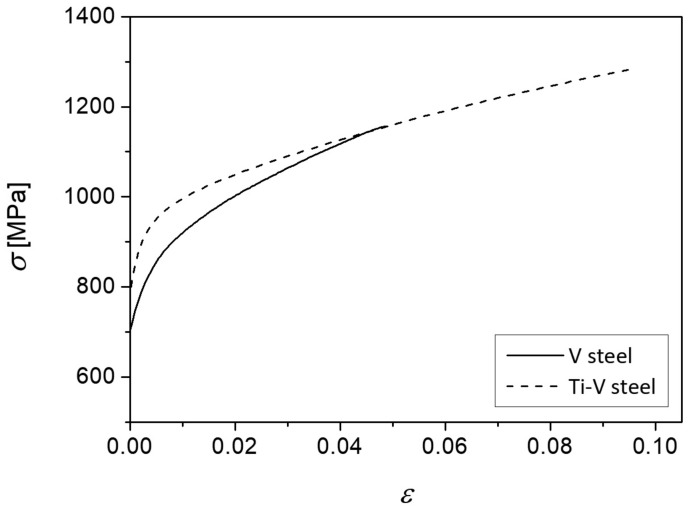
Fitted experimental tensile true stress–true strain curves of the V steel and the Ti-V steel samples tested at −196 °C.

Plastic yielding is confined within the narrow zone, about 1.5 mm from the notch root for V steel, and about 0.75 mm for Ti-V steel (Figure 5 and Figure 6). Plastic strains in V steel reach values as high as 0.6, while in Ti-V steel they are limited to much lower values, up to approximately 0.15 (Figure 5, Table 3). In the same manner, the values of the plastic strains at the cleavage fracture initiation sites, *ε*_pc_, given in Table 3, are also considerably different for the two steels examined. Observed behavior could be related to the stress–strain response of the steels, presented by the fitted experimental true stress–strain curves at −196 °C, shown in Figure 7 [50], which had been used as a model of the mechanical properties in FEM. Ti-V steel exhibits considerable ductility at −196 °C in comparison with V steel sample. On the other hand, V steel is characterized by the higher strain-hardening rate, leading to the observed distribution of the plastic strain around the notch and formation of the narrow plastic zone with steeper strain increase toward the notch in V steel than in Ti-V steel four-point bending sample. The deformation behavior of the steels tested could be related to its microstructure and the content of the individual microconstituents. It is known that the increase of the pearlite volume fraction leads to the increase of the strength and strain-hardening rate [51,52].

Further, both steels exhibit a gradual- or continuous-yielding effect, which manifests as the relatively low YS, quantified as the *R*p_0.2_value, followed by the steep stress increase at low strains (Figure 7), and the consequently lower YS/UTS ratio. This behavior is characteristic of the steels with bainitic or AF structure that contains high density of mobile dislocations due to a displacive nature of transformation [53,54]. Furthermore, another possible cause for the observed mechanical response could be the presence of the retained austenite, characteristic of the medium-carbon microalloyed steels with bainitic or AF structure, related to the incomplete reaction phenomenon [55]. It could be in particular related to the observed tensile elongation of the Ti-V steel specimens at such low temperatures (Figure 7). In conclusion, the observed distribution of the strain in the four-point bending specimen, calculated by FEM, was a consequence of the mechanical behavior of the continuously cooled steel microstructures with the predominant content of AF and the contribution of the various amount of pearlite and GBF depending on the cooling rate as well as the chemical composition of the microalloyed steels.

Having in mind that the origins of the cleavage fracture were found exclusively in the narrow area at the notch root, it could be assumed that high plastic deformation led to the formation of the microcracks by fracture of the microconstituents with low ductility, primarily the pearlite, which resides in the microstructure between the AF and GBF. A rupture of GBF grains by the Smith’s mechanism due to low ductility at −196 °C should also be taken into account. The size of the facets at the cleavage origin, determined as the effective diameter, D_eff_, shown in Table 3, is rather similar for V and Ti-V steel, with the average values of 17 μm and 19 μm, respectively. A similar size of the first facet’s diameter regardless of the size and the volume fraction of pearlite and GBF in the two steels may imply a possibility that initial microcracks form also in AF, presumably at sheaves of ferrite plates with similar crystallographic orientation (lower AF).

However, distances of the cleavage origins from the notch in Ti-V steel 4PB specimens are somewhat larger, and therefore the local cleavage stress is higher. Although nominal fracture stress, *σ*_F_, is lower for the Ti-V steel (Table 3, columns 2 and 3), the local stress at the initiation site is higher than in the V steel. Therefore, when calculated using Griffith’s equation for the circular crack (Equation (1)), the value of the effective fracture surface energy is noticeably higher for the Ti-V steel (Table 3, column 10).

**Figure 8:**
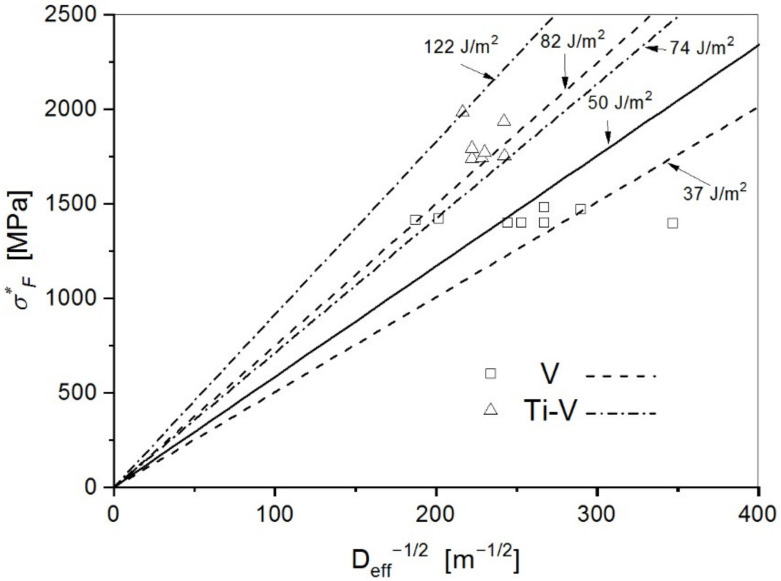
Values of the local critical fracture stress plotted against the reciprocal square root of the first cleavage fracture facet’s effective diameter and the corresponding values of the effective fracture surface energy.

Considering linear dependence of the local critical fracture stress, *σ*_F_^*^, on the reciprocal square root of the initial microcrack effective diameter, D_eff_^−1/2^, in accordance with the Griffith’s equation (Equation (1)) lines have been drawn in the graph in Figure 8 in order to calculate the values of the effective surface energy for cleavage fracture, *γ*. Experimental points lie in the range between 37 Jm^−2^ and 82 Jm^−2^ for the V steel and between 74 Jm^−2^ and 122 Jm^−2^ for the Ti-V steel. From the line drawn just below the lowest experimental point, an assumed upper value of the true effective surface energy for cleavage fracture, *γ*, is calculated. Therefore, the values of 37 Jm^−2^ and 74 Jm^−2^ could be adopted for the V and Ti-V steel, respectively. One of the experimental points for V steel deviates to a large degree, giving the unrealistic value of 24 Jm^−2^, and therefore it was excluded from the analysis. This point matches the unusually small facet diameter in sample 3 in Table 3. Having in mind that cleavage initiation, in this case, is not related to the fracture of particles but to the plastic deformation at the notch root, the calculated values of the effective surface energy for cleavage fracture could be considered as the values for the propagation of the crack through the grain boundaries, *γ = γ*_mm_. In that manner, it indicates the role of the fine interlocking structure of AF in the cleavage fracture mechanism. The value of the effective fracture surface energy of 37 Jm^−2^ for the V steel with a mixed microstructure consisting of about 70% of AF, and the rest being mostly the pearlite and GBF, is comparable to the values for the medium-carbon microalloyed steels with the ferritic-pearlitic and bainitic structures found in the literature [20,23].

However, the value 74 Jm^−2^ for the Ti-V steel is considerably higher, and it could be assumed that the observed increase in the effective surface energy was due to the higher density of high-angle boundaries of the AF structure. Additionally, another rather indirect effect of the AF through its influence on the stress–strain behavior and resultant stress and strain distribution in front of the notch in 4PB specimen could be considered.

First, due to the characteristic high dislocation density, the observed low yield strength or the “gradual yielding” effect, alongside with the relatively high ductility at low temperatures (Figure 7), induce high plastic strains at relatively modest stress levels near the notch, which cause the fracture of the favorably oriented pearlite nodules or coarse eutectoid ferrite grains.

Formed microcracks are large enough to trigger the cleavage fracture at low stresses near the notch root. At the same time, peak stress at the distance *X*_0_, 400–500 µm from the notch root, had not been sufficient for the cleavage initiation by fracture of the coarse TiN particles. Taking the value of γ_pm_ =7 Jm^−2^for the fracture of the coarse TiN particles [40,56], calculation using Griffith’s equation (Equation (1)) gives the value of approximately 2500 MPa for the grain sizes smaller than 5.45 μm for V steel and 7.85 μm for Ti-V steel. Peak values of the maximum principal stress, *σ*_1max_, for the V steel samples are in the range 2595–2671 MPa, while for the Ti-V steel samples are considerably lower, from 2139–2430 MPa (Table 3). Therefore, it follows that the peak values of the maximum principal stress, σ_1max_, in this work was lower than the stress needed for the fracture of the coarse TiN particles in Ti-V steel. As regards V steel it could not be expected a significant number of large TiN particles considering the low content of Ti (Table 1). However, in previous researches, it was noticed that TiN particles could not trigger the cleavage fracture when embedded in a ductile and fine grained matrix [47,57]. The results in this investigation confirm that TiN remains neutral regarding the cleavage fracture initiation in the fine interlocking structure of acicular ferrite. In conclusion, the large cracks formed in the zone of high plastic deformation trigger the cleavage fracture before the critical conditions are met for the cleavage initiation by fracture of the coarse TiN particles in the zone of peak stress.

Furthermore, it also indicates the role of the pearlite and ferrite in the cleavage fracture mechanism of the microalloyed steels with predominantly AF structure as potential sites for the formation of microcracks. Therefore, a lower number of perlite nodules and polygonal ferrite grains in Ti-V steel implicate fewer sites for cleavage initiation. It could also explain somewhat larger distances of the cleavage fracture origin from the notch in Ti-V steel. Conversely, a larger number of pearlite nodules and proeutectoid ferrite grains in V steel represent a larger number of potential sites for the cleavage fracture initiation at lower stress levels near the notch root. While the fine interlocking structure of AF contributes to the increase of the local critical fracture stress, low-ductility coarse microconstituents–pearlite, GBF, or ferrite-pearlite aggregates with the same crystallographic orientation decrease the critical cleavage fracture stress, considering that they are suitable sites for cleavage fracture initiation, at relatively low stresses, in the medium carbon microalloyed steels with predominantly AF structure.

## 4. Conclusions

1. Cleavage fracture of air-cooled medium carbon microalloyed steels with predominantly acicular ferrite microstructure is initiated by the fracture of the coarse microstructural units, ferrite, and pearlite, under the plastic deformation at the notch root of the four-point bending specimen. In this case, fracture of the second phase particles is not involved in the cleavage fracture initiation process.
2. Peak stress in front of the notch root of the four-point bending specimen is insufficient for the cleavage fracture initiation by fracture of coarse TiN particles in the microstructure consisting predominantly of acicular ferrite due to its fine-grained structure with a high density of high-angle boundaries and relatively low strength and considerable ductility at liquid nitrogen temperature, comparing to the ferritic-pearlitic structure.
3. While the coarse ferrite grains and pearlite nodules govern the cleavage fracture initiation, stress and strain distribution in the four-point bending specimen are dominated by the acicular ferrite and its deformational characteristics. It is assumed that mechanical properties were also contributed by the retained austenite present in the acicular ferrite structure of the steel with higher carbon content and the Ti-V microalloying addition.
4. Estimated values of the effective surface energy for the V steel with about 70% of the acicular ferrite of 37 Jm^−2^, and for the Ti-V steel with the acicular ferrite content as high as 96% of 74 Jm^−2^ are considered as the effective energy for the propagation of the crack through the grain boundaries, and therefore directly relate to the effect of the fine interlocking structure of acicular ferrite in the cleavage fracture mechanism.

## Figures and Tables

**Table 1:** Chemical composition of the steels (wt.%).

Steel	C	Si	Mn	P	S	Cr	Ni	Mo	V	Ti	Al	Nb	N
**Ti-V**	0.309	0.485	1.531	0.0077	0.0101	0.265	0.200	0.041	0.123	0.011	0.017	0.003	0.0228
**V**	0.256	0.416	1.451	0.0113	0.0112	0.201	0.149	0.023	0.099	0.002	0.038	0.002	0.0229

**Table 2:** Redistribution of the Ti, V, and N elements between particles and solid solution, and temperatures for the complete dissolution of VN and VC precipitates.

**Steel**	**[Ti]** **[ppm]**	**[N]** **[ppm]**	**[N]TiN** **[ppm]**	**[N]VN** **[ppm]**	**[V]** **[wt.%]**	**[V]VN** **[wt.%]**	**[V]excess** **[wt.%]**	**T_VN_** **[°C]**	**T_VC_** **[°C]**
Ti-V	110	228	32	196	0.123	0.071	0.052	1117	894
V	20	229	6	223	0.099	0.081	0.018	1108	869

**Table 3:** Critical parameters for the cleavage fracture initiation for V and Ti-V steel samples.

V Steel Sample	σ_F_ [MPa]	σ_F_/σ_0_	σ_1max_ [MPa]	X_0_ [μm]	σ_F_* [MPa]	ε_pc_	D_max_ × D_min_ [μm]	D_eff_ [μm]	γ [Jm^−2^]
1	1193	1.54	2645	32	1419	0.493	36.8 × 15.5	24.5	72.3
				53	1471	0.450	12.8 × 17.3	11.9	37.7
2	1268	1.64	2671	19	1400	0.634	23.7 × 9.8	15.6	44.7
				19	1400	0.634	21.0 × 11.3	16.7	48.0
				28	1414	0.616	29.3 × 24.1	28.5	83.7
3	1049	1.35	2595	15	1398	0.369	41.0 × 8.1	14.0	40.1
				14	1396	0.370	8.6 × 6.8	8.3	23.6
4	1152	1.49	2640	56	1479	0.409	34.7 × 8.2	14.0	45.1
**Ti-V Steel Sample**	**σ_F_** **[MPa]**	**σ_F_/** **σ_0_**	**σ_1max_** **[MPa]**	**X_0_** **[** **μm]**	σ_F_* **[MPa]**	**ε_pc_**	**D_max_ × D_min_** **[** **μm]**	**D_eff_** **[** **μm]**	**γ** **[Jm^−2^]**
1	759	0.85	2139	78	1739	0.060	20.4 × 18.3	20.2	89.7
2	934	1.04	2402	59	1751	0.152	18.8 × 12.7	17.0	76.5
				56	1740	0.154	22.3 × 13.6	19.1	85.0
3	904	1.01	2343	112	1934	0.092	17.2 × 15.3	17.0	93.4
				131	1983	0.082	22.1 × 17.6	21.3	123.0
4	950	1.06	2430	71	1792	0.156	35.4 × 12.3	20.2	95.2
				65	1770	0.161	29.1 × 11.8	18.8	86.6

## Data Availability

The data presented in this study are available on request from the corresponding author. The data are not publicly available.
